# Supplementary material for: One-Pot Synthesis, E-/Z-Equilibrium in Solution of 3-Hetarylaminomethylidenefuran-2(3H)-ones and the Way to Selective Synthesis of the E-Enamines
Source: Molecules. 2023 Jan 18;28(3):963. doi: 10.3390/molecules28030963 (PMC9921198; doi:10.3390/molecules28030963)

Supplementary Materials

One-Pot Synthesis, *E*-/*Z*-Equilibrium in Solution of 3-Hetarylaminomethylidenefuran-2(3*H*)-ones and the Way to Selective Synthesis of the *E*-Enamines

Alexandra S. Tikhomolova [1,*], Vyacheslav S. Grinev [1,2] and Alevtina Yu. Yegorova [1]

[1] Institute of Chemistry, N.G. Chernyshevsky Saratov National Research State University, 83 Ulitsa Astrakhanskaya, Saratov 410012, Russia; grinevvs@sgu.ru (V.S.G.); yegorovaay@gmail.com (A.Y.Y.);

[2] Institute of Biochemistry and Physiology of Plants and Microorganisms—Subdivision of the Federal State Budgetary Research Institution Saratov Federal Scientific Centre of the Russian Academy of Sciences (IBPPM RAS), 13 Prospekt Entuziastov, Saratov 410049, Russia

* Correspondence: bondartsova.alexandra@yandex.ru

Table of content

The ^{1}H & ^{13}C, NOESY NMR spectrum of compound **9a** S2

The ^{1}H NMR spectrum of compound **9b** S3

The ^{1}H & ^{13}C NMR spectrum of compound **9c** S4

The ^{1}H & ^{13}C NMR spectrum of compound **9d** S5

The ^{1}H & ^{13}C NMR spectrum of compound **9e** S6

The ^{1}H & ^{13}C NMR spectrum of compound **9f** S7

Figure S1. ^{1}H & ^{13}C NMR of **9a**

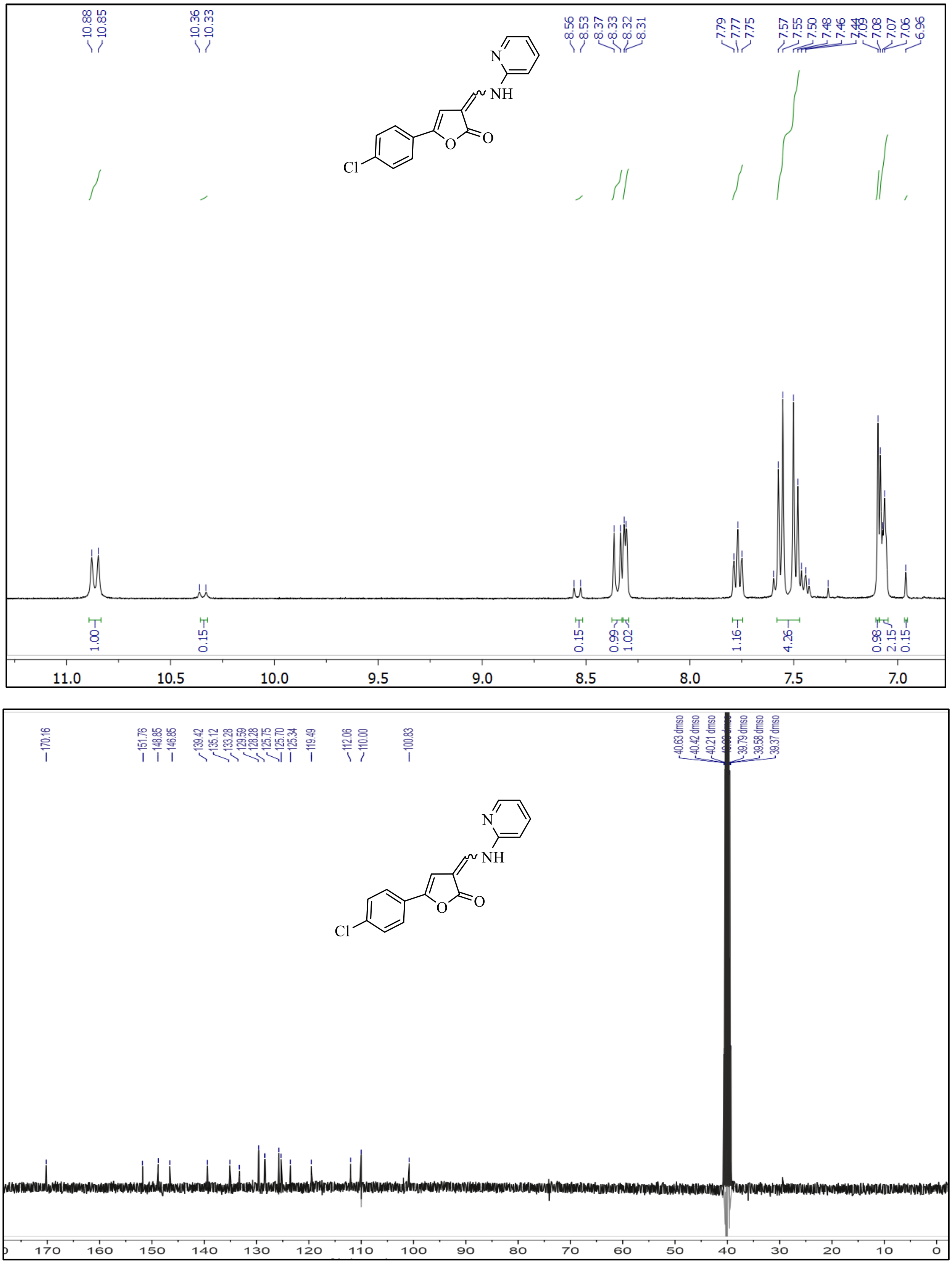

Figure S2. NOESY of **9a**

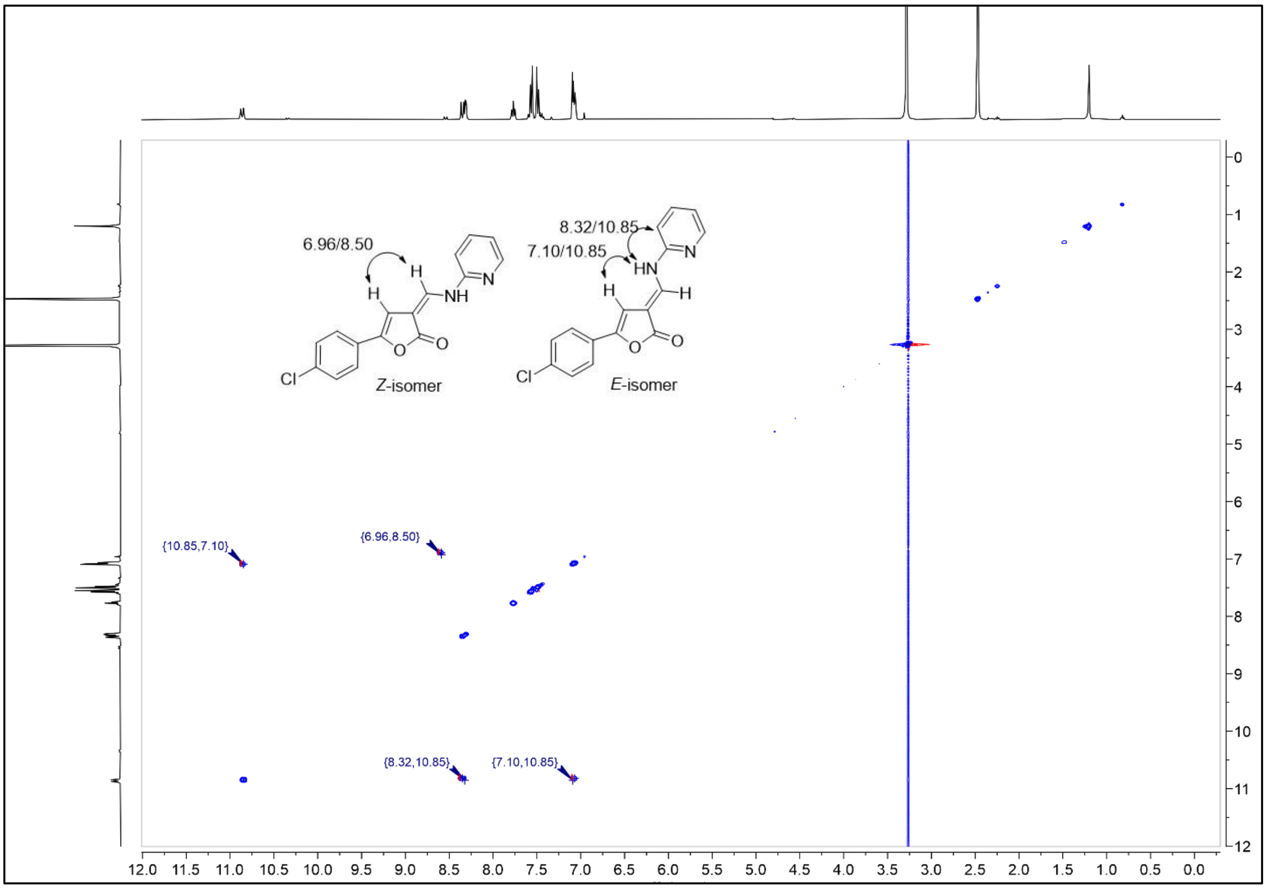

^{1}H NMR of **9b**

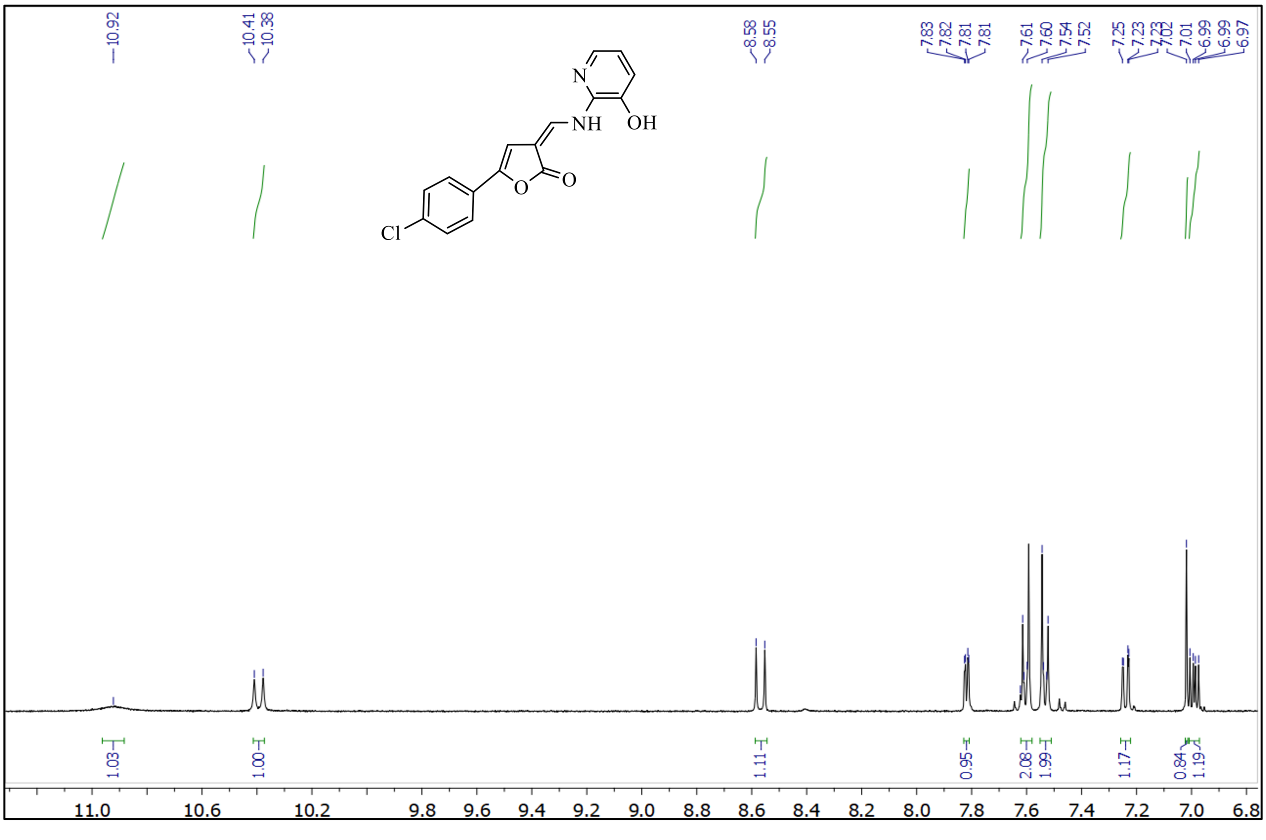

Figure S3. ^{1}H & ^{13}C NMR of **9c**

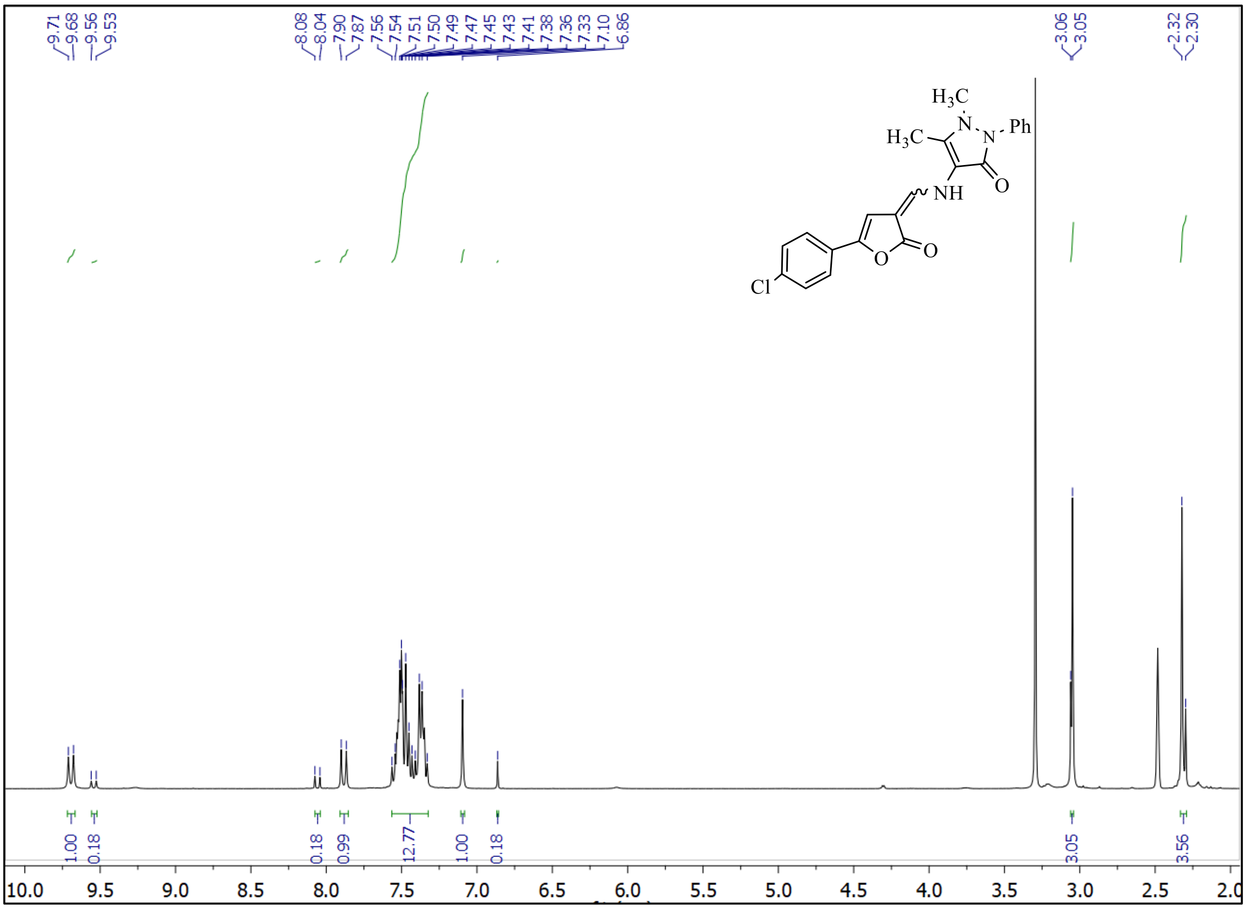

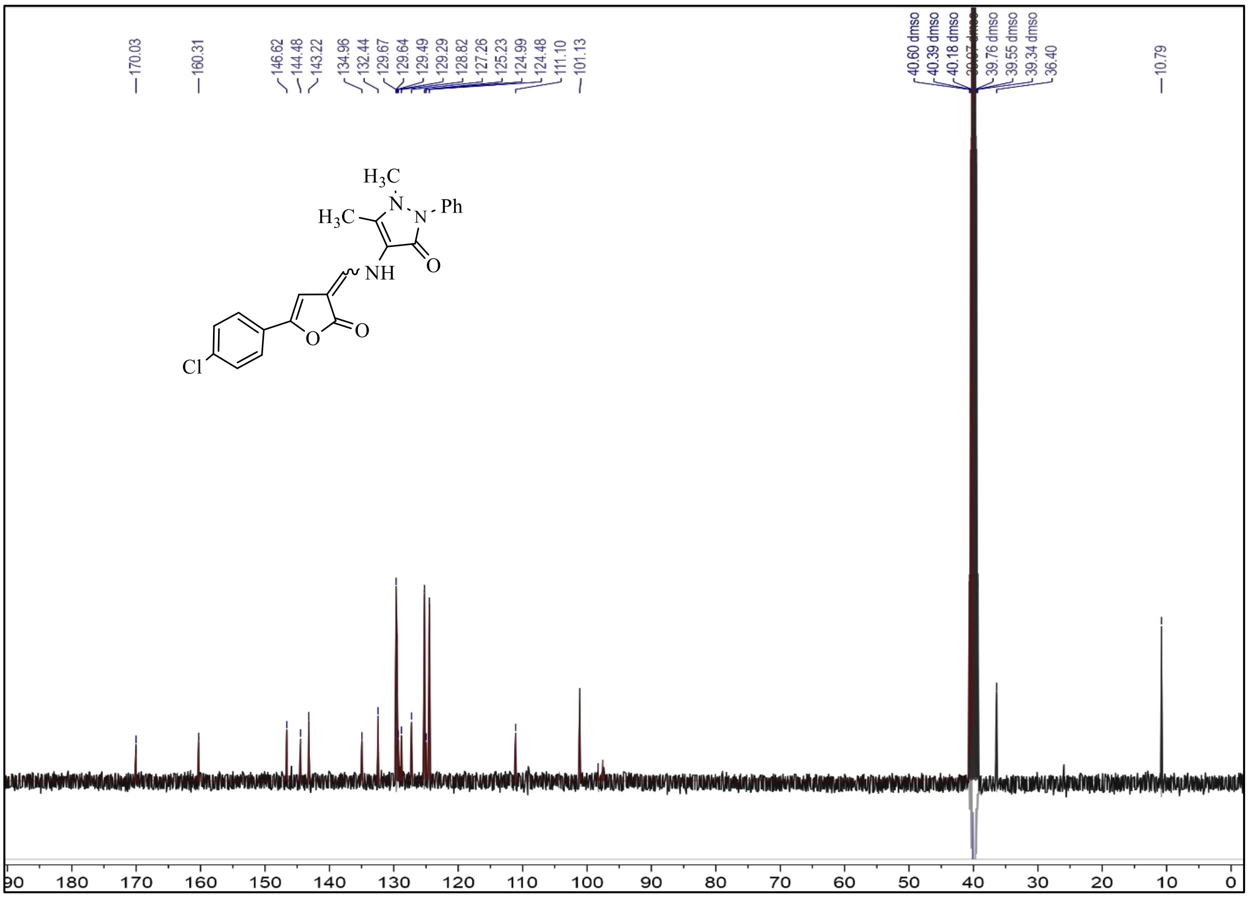

Figure S4. ^{1}H & ^{13}C NMR of **9d**

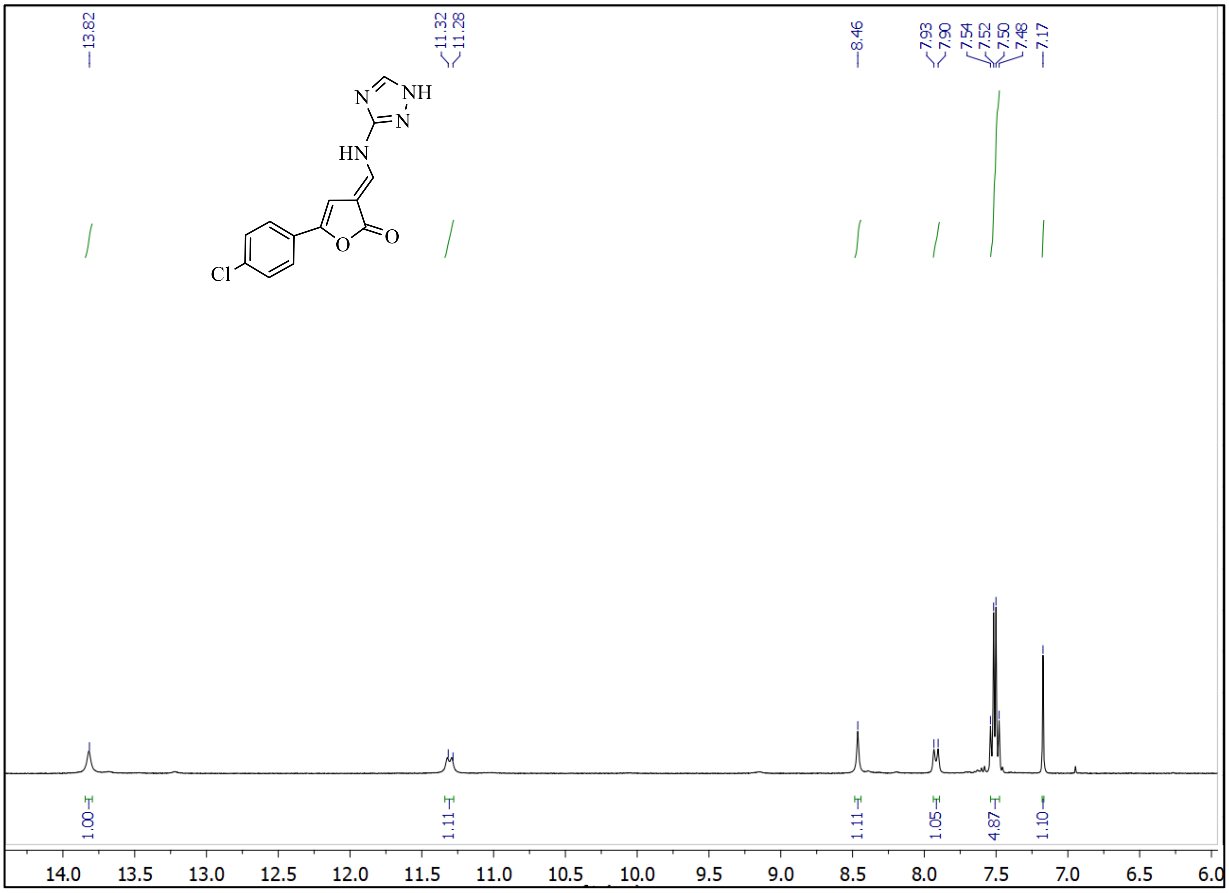

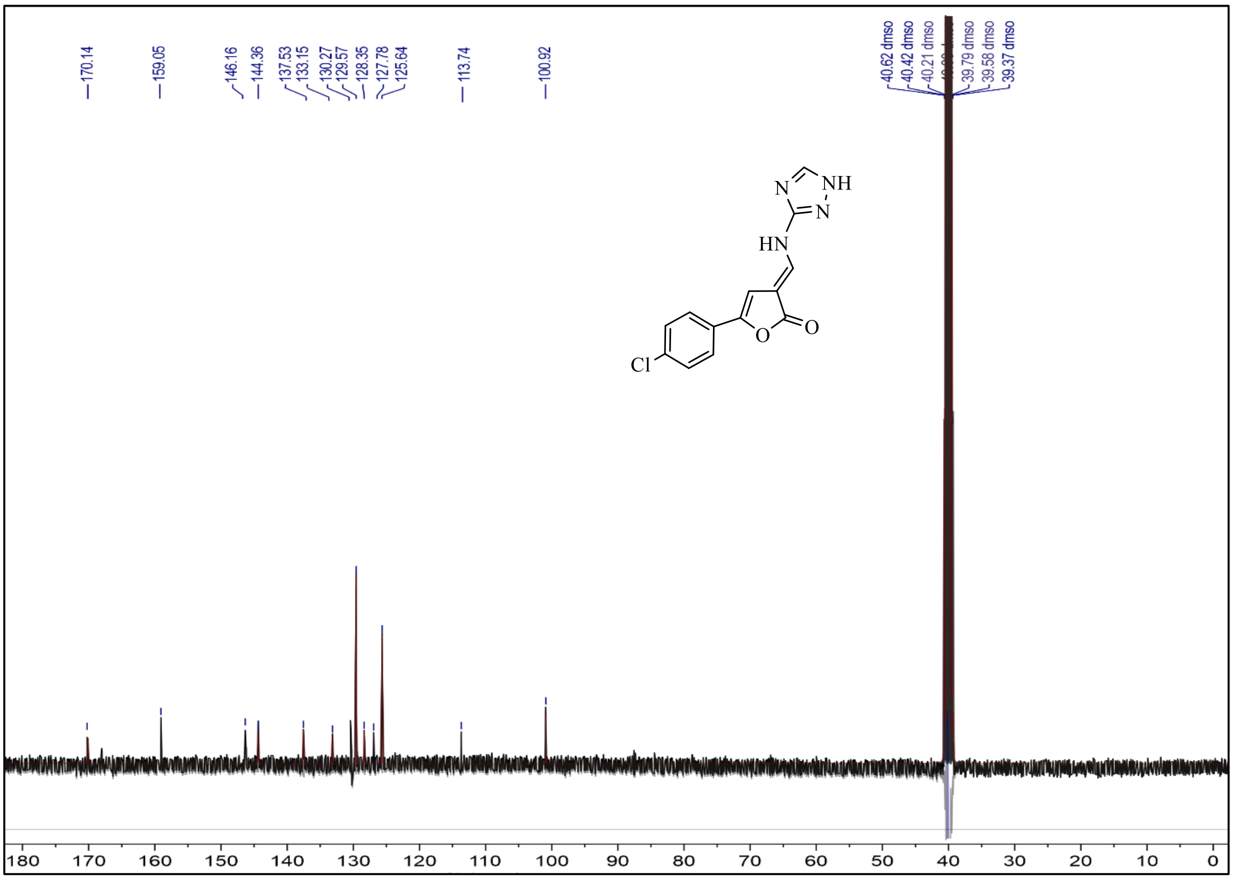

Figure S5. ^{1}H & ^{13}C NMR of **9e**

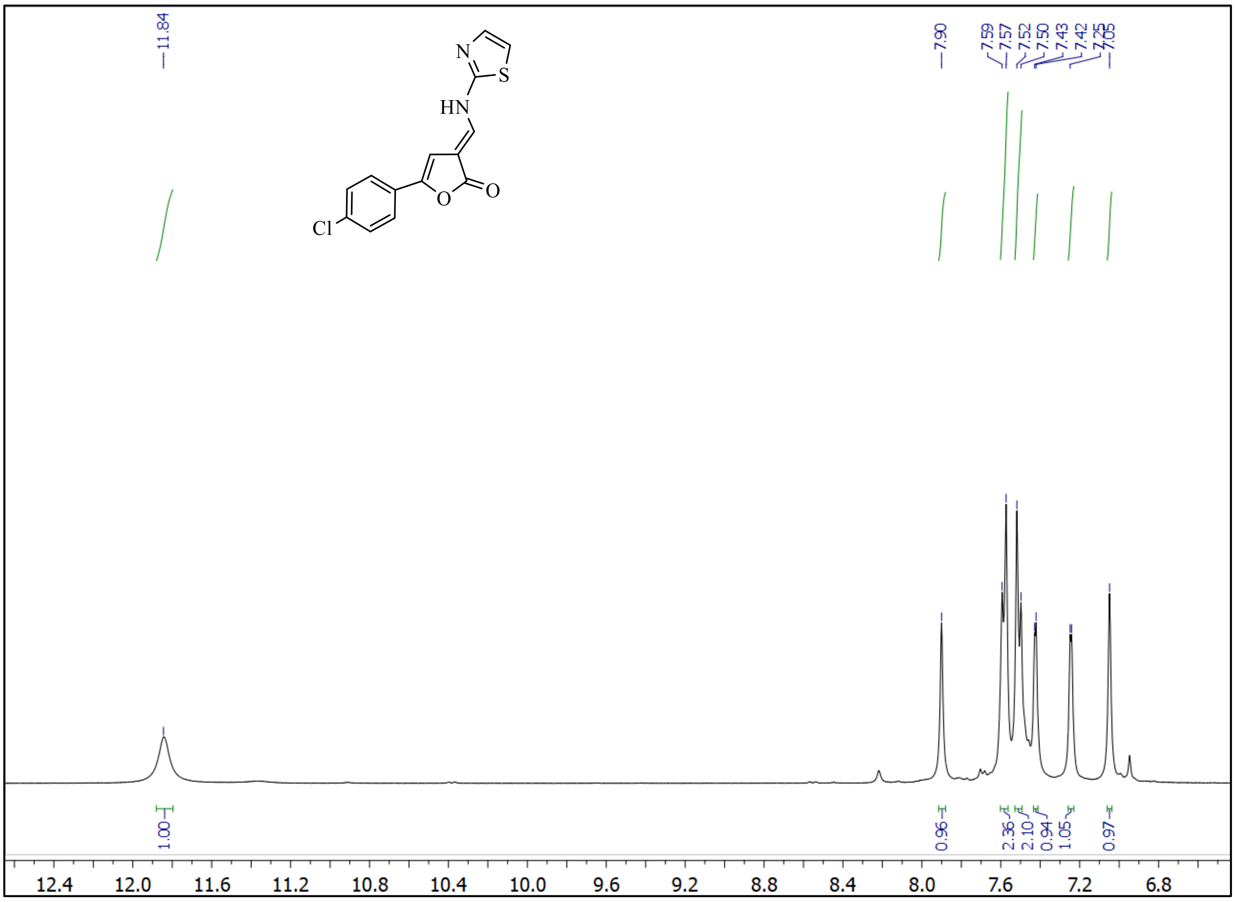

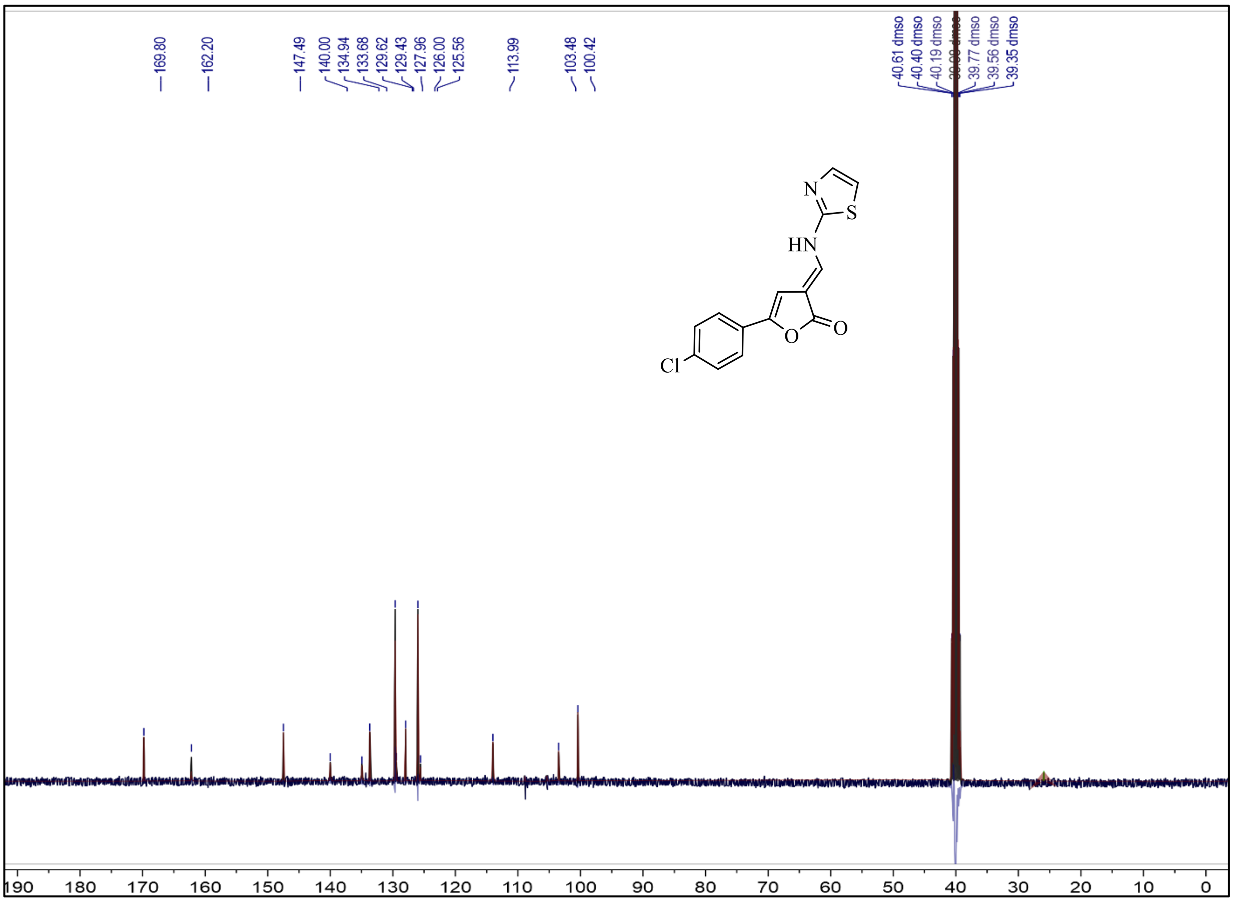

Figure S6. ^{1}H & ^{13}C NMR of **9f**

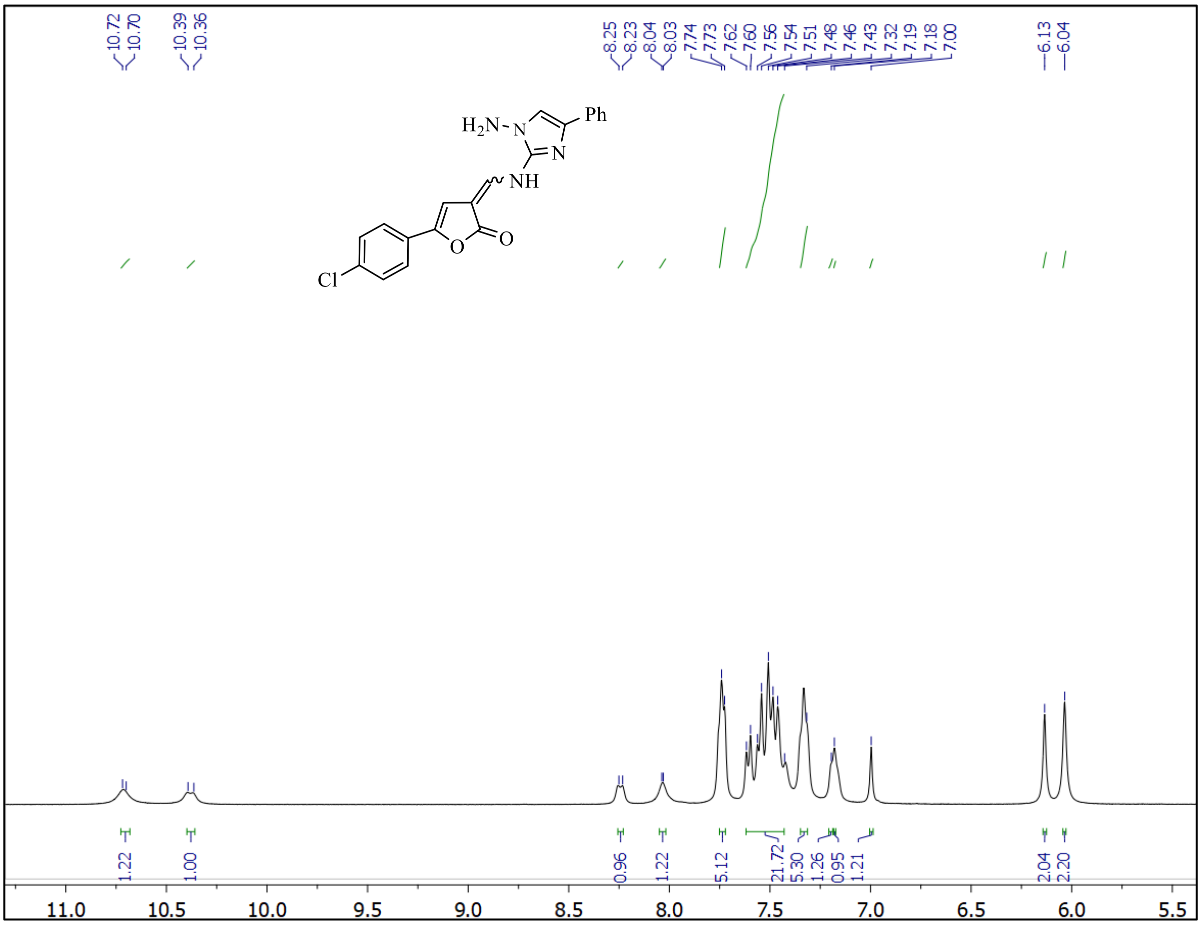

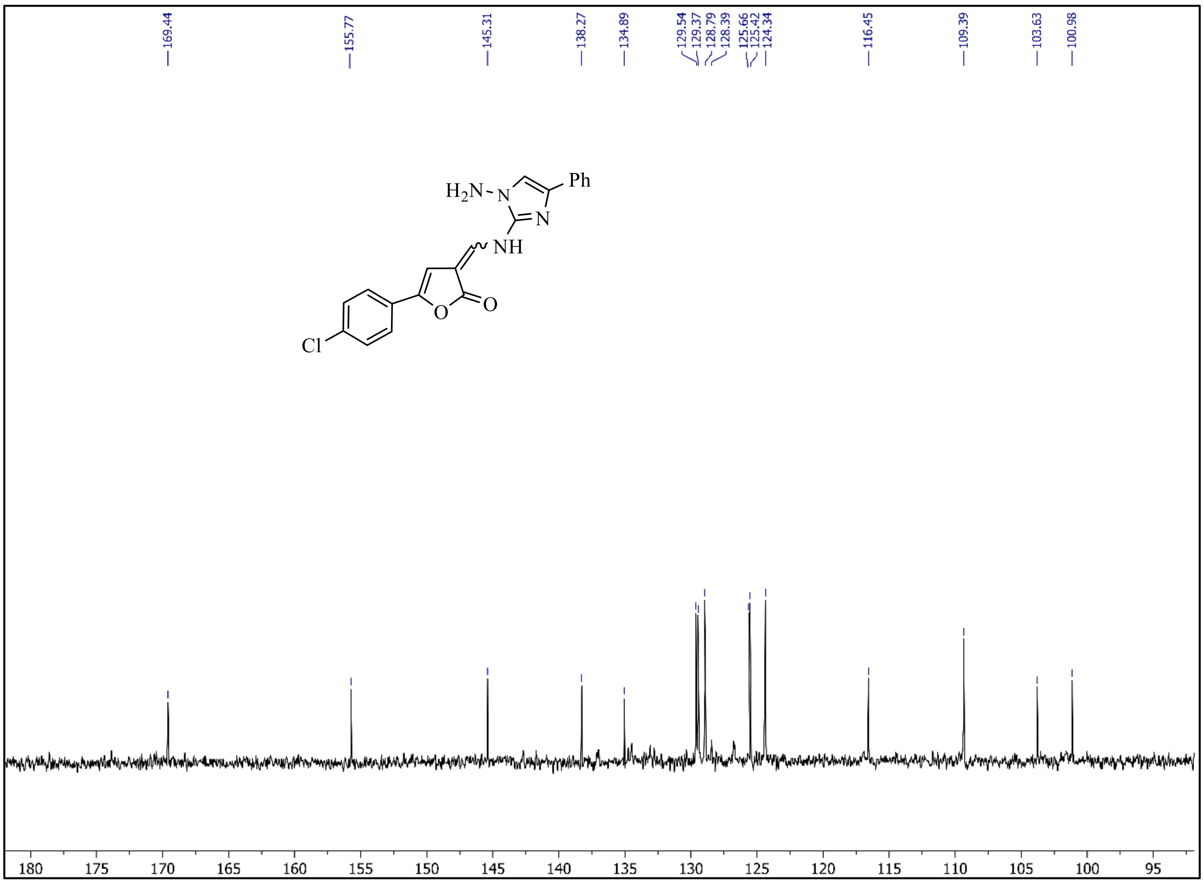

Supplement: Supplementary file 1 [file molecules-28-00963-s001.zip › molecules-2106138-supplementary.pdf]
